# Insight into the Roles of Albumin—Alone and in Combination with Either Voriconazole or Antimicrobial Peptides Derived from Chromogranin A—In the Growth of Different Microbial Species

**DOI:** 10.3390/antibiotics14100974

**Published:** 2025-09-26

**Authors:** Francis Schneider, Sophie Hellé, Jean-Marc Strub, François-Xavier von Hunolstein, Pierre Schaaf, Philippe Lavalle, Francesco Scavello, Marie-Hélène Metz-Boutigue

**Affiliations:** 1 INSERM UMR_S 1121, CNRS EMR 7003, Université de Strasbourg, Biomaterials and Bioengineering, Centre de Recherche en Biomédecine de Strasbourg, 1 rue Eugène Boeckel, F-67000 Strasbourg, France; francis.schneider11@wanadoo.fr (F.S.); s.helle@unistra.fr (S.H.); fxvonhunolstein@yahoo.fr (F.-X.v.H.); pierre.schaaf@inserm.fr (P.S.); philippe.lavalle@inserm.fr (P.L.); 2 Federation of Translational Medicine, Faculty of Medicine, Strasbourg University, 67081 Strasbourg, France; 3 Laboratoire de Spectrométrie de Masse BioOrganique, Strasbourg University, CNRS IPHC UMR 7178, 67081 Strasbourg, France; jmstrub@unistra.fr; 4 IRCCS Humanitas Research Hospital, 20089 Rozzano, Milan, Italy

**Keywords:** albumin, *Aspergillus*, *Candida*, cateslytin, catestatin, chromogranin A, infection, voriconazole

## Abstract

Background: Whether therapeutic albumin (ThHSA) can serve as a defense tool in *Candida* species (spp.) infections is still a matter of debate, although many physicians are in the habit of infusing ThHSA to restore the physiological concentration of endogenous human serum albumin (HSA). Given the need for innovative anti-Candida strategies, we assessed *in vitro* the role of ThHSA alone or in combination with voriconazole (VCZ) in combating *Candida* spp. growth and the role of bovine serum albumin (BSA)—used as a substitute for HSA—with two endogenous bovine antimicrobial peptides in combating *C. albicans* and other microbes. Results: The combination of ThHSA with VCZ enhanced the antifungal effect on *C. albicans*, sensitive *C. tropicalis,* sensitive *C. glabrata*, and *C. lusitaniae*. However, for resistant *C. tropicalis*, the combination of ThHSA with VCZ promoted yeast growth, and VCZ tended to suppress the antimicrobial effect of ThHSA on resistant *C. glabrata*. As to the possible transposition of ThHSA-type properties to BSA (as regards the growth inhibition of other pathogens), we tested combinations of BSA with two physiological chromogranin A-derived antimicrobial peptides (catestatin and cateslytin). BSA enhanced significantly the activity of catestatin (but not cateslytin) in combating *C. albicans, A. fumigatus,* and *M. luteus,* but was inactive against *S. aureus* and *E. coli*. Conclusions: Our experiments support the fact that albumins display intrinsic antimicrobial properties, with an unpredictable growth inhibitory effect on various microbes. ThHSA can thus be an adjunctive tool for more efficient care of some, though not all, infections. The interaction of BSA with catestatin and cateslytin is related to their structure, with BSA significantly enhancing the effect of catestatin but not that of cateslytin.

## 1. Introduction

*Candida* spp. infections are common in fragile patients. Invasive candidiasis is the fourth leading cause of bloodstream infections worldwide, with mortality rates as high as 40% [1]. Recently *Candida* spp. were identified as the third most frequently isolated pathogen, accounting for 17% of all infections in intensive care units [2,3]. The ability of *Candida* spp. to colonize implanted medical devices [4] further contributes to the occurrence of nosocomial infections (NIs) [5,6]. While *C. albicans* is involved in approximately two-thirds of invasive candidiasis cases, nowadays non-albicans *Candida* spp. (*C. glabrata*, *C. krusei*, *C. tropicalis,* and *C. parapsilosis*) account for nearly half of all infections [1]. Furthermore, the effectiveness of antifungal therapy in relation to these strains also appears to be on the decrease, because (1) if microbiological methods for diagnosing candidemia are sub-optimal, they lead to the delayed infusion of the appropriate antifungal therapy, thereby increasing mortality [7,8]; (2) unintentional sub-optimal antifungal dosing administration is linked to the development of treatment resistance [9]. Among available antifungal agents, amphotericin B and echinocandins are recommended for their potent activity in combating *Candida* spp., but such effectiveness is now threatened by the emergence of drug-resistant *Candida* strains [10]. This situation may require a therapeutic switch to voriconazole (VCZ), a triazole antifungal agent with proven efficacy in invasive infections [11], even triggered by some *C. glabrata* and *C. krusei* [12].

A recent pilot study has underscored that ThHSA is associated with a lesser occurrence of NIs in patients with septic shock (including *Candida* spp. infections) provided that ThHSA is run as a continuous low concentration infusion [13]. Albumin is a globular protein with critical physiological roles, including the transport of a wide range of exogenous and endogenous ligands [14]. Hypoalbuminemia is common and ominous in patients with shock [15]. Physicians have therefore taken to infusing therapeutic albumin (ThHSA) in an attempt to restore the physiological concentrations of endogenous HSA, although clinical trials differ as to the relevance of pursuing such an objective [16]. Critically ill patients are commonly infused with ThHSA for (1) its oncotic power, which helps in restoring shock-induced circulatory failure [17]; and (2) its association with improved outcome in selected patients [13,16]. Albumin interacts with antimicrobial peptides, modifying their action through binding at specific drug sites [18,19,20]. A new thiazole derivative binds albumin to reach the target site [21], and a complex amphotericin–albumin is highly effective in combating *C. albicans*, with lower toxicity for patients [22]. In a previous study, it was also reported that the three albumins (human, bovine, and ovalbumin) display antimicrobial activities against different microbial cultures and more specifically *C. albicans*; this effect was dose-dependent and a lytic effect was demonstrated with the formation of debris vesicles and the destruction of microbial membranes [23]. A recent study on *C. albicans* infection underscores how albumin reduces host cell damage by neutralizing candidalysin [24]. Additional experimental arguments for its benefit in severe infection were that ThHSA reverses defective vasomotion [25]. Finally, in an observational clinical trial designed to assess the impact of continuous low doses of ThHSA in patients with septic shock, there was a significant decrease in care-related colonization and infection (including by *Candida* spp.) [26]. Altogether these data strongly suggest that albumin may serve as a defense tool against *Candida* infection.

Given the crucial need for innovative fungicide strategies to combat both infection and resistance and, also, to prevent NI occurrence, we decided to assess *in vitro* the potential role of albumins in *Candida* spp. infections. We therefore investigated the *in vitro* impact of (1) ThHSA on *Candida* spp. growth alone and in association with VCZ; and (2) bovine serum albumin (BSA) on the antifungal activity of two endogenous antimicrobial peptides derived from the processing of chromogranin A (CgA), namely catestatin (CTS) and cateslytin (CTL) [27].

In a previous study, we reported the following data: (1) the most active peptide in combating strains of *C. albicans*, *C. tropicalis*, and *C. glabrata* was D-bCTL, the D-isomer of CTL (CgA344-358) at low concentrations, whereas the efficiency of the L-isomer (L-bCTL) significantly decreased against *C. glabrata* strains [20]; (2) L-bCTL was able to kill *C. albicans* with a concentration of 7.9 µg/mL, inducing a lytic effect, whereas L-bCTS (CgA344-364) was active at 30 µg/mL, suggesting that the C-terminal sequence (GPGLQL) of CTS may interact with the membrane to prevent penetration into *C. albicans* [20]; (3) quartz crystal microbalance (QCM) analysis demonstrated that BSA interacts with CTS but not CTL, which indicates that the conformation of CTS with the C-terminal sequence (GPGLQL) is important for the interaction with BSA; (4) BSA (0.4 ng/mL, 1.6 ng/mL) enabled the penetration of CTS (9.6 µg/mL) into *C. albicans*, but the positive effect of BSA was not observed for the antimicrobial activity of CTL [20]. In the present study, for CTS and CTL, growth inhibition tests were performed on additional microbes (bacteria and fungi) so as to understand whether the concept was transposable.

## 2. Results

### 2.1. Antimicrobial Assays with ThHSA Alone or in Combination with VCZ

The experimental design of this study is illustrated in Figure S1.

The *Candida* strains used in the present study were characterized on the basis of the Etest method as described in Section 4 and in a previous study by the Institute of Parasitology and Tropical Pathology in Strasbourg [20]. Among the strains susceptible to fluconazole *C. albicans S* (1610m08105/04), *C. tropicalis S* (1601m110335), *C. glabrata S* (1606m050098), and *C. lusitaniae* (1601m140864/02), VCZ exhibited the highest activity, with the lowest minimum inhibitory concentrations (MICs) recorded at 0.002 µg/mL, 0.047 µg/mL, 0.032 µg/mL, and 0.012 µg/mL, respectively. For the resistant strains *C. albicans R* (1610m100288), *C. tropicalis R* (1602m010892), and *C. glabrata R* (1507m150549), caspofungin demonstrated the lowest MIC values, measured at 0.047 µg/mL, 0.125 µg/mL, and 0.250 µg/mL, respectively. We tested the ability of the combination VCZ/albumin to fight these resistant strains.

The purity of HSA and ThHSA was assessed using nano high-performance liquid chromatography (HPLC) on a Vydac 208TP C8 column, with peak detection at an absorbance of 214 nm, as reported in Section 4. Both HSA and ThHSA co-eluted at 35 min (Figure S2A). The theoretical molecular mass of unmodified HSA is 66,472.21 Da (UniprotKB P02768, residues 25-609). Mass spectrometric analysis of the HSA and ThHSA peaks was performed using a hybrid electrospray quadrupole time-of-flight instrument, as detailed in Section 4. The observed masses were 66.463 Da for HSA and 69.240 Da for ThHSA (Figure S2B).

**Figure 1:**
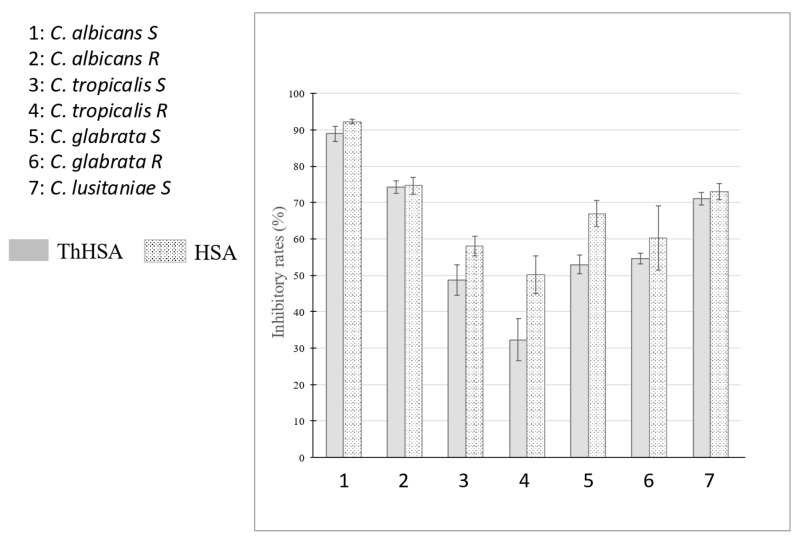
Antimicrobial activities of ThHSA and HSA (20 mg/mL) in combating sensitive (S) and resistant (R) *Candida* spp. Microbial growth was quantified by measuring absorbance at 620 nm. Positive control corresponds to VCZ and negative control to MilliQ water. The inhibitory rates (%) were evaluated in triplicate. Data are presented as mean +/− standard deviation.

As a start, we analyzed the *in vitro* impact of both ThHSA (Vialebex, 200 mg/mL, LFB, Les Ulis, France) and its reference chemical standard (HSA, 20 mg/mL, purchased at Bio-Rad, Marnes-La-Coquette, France) on the growth of seven *Candida* spp., some being sensitive (S) and others resistant (R) to fluconazole (Figure 1).

The concentrations of ThHSA and HSA (20 mg/mL) used in this section were selected on a relevant concentration basis for patients with shock at intensive care department admission, that is at a dilution of ThHSA (200 mg/mL) within circulation. Such a circulating concentration is met frequently for albumin in the critically ill, and running ThHSA at standard dosages (60 g a day of 20% ThHSA) or at low dosages (150 mg/kg of a-day 4% ThHSA) can restore circulating albumin in the range of 25–30 g/L over five days of treatment, as reported elsewhere [21]. For several strains, ThHSA and HSA exhibited similar inhibitory effects, as illustrated for *C. albicans* S (88.9% and 92.3%), *C. albicans* R (74.3% and 74.7%), *C. glabrata* R (54.6% and 60.3%), and *C. lusitaniae* S (71.0% and 73.1%) (Figure 1). These findings suggest that the antimicrobial activity is directly associated with HSA. However, differences were observed for *C. tropicalis* S (48.7% vs. 58.1%), *C. tropicalis* R (32.3% vs. 50.1%), and *C. glabrata* S (52.9% vs. 67.0%), indicating a variable response for the latter strains.

**Figure 2:**
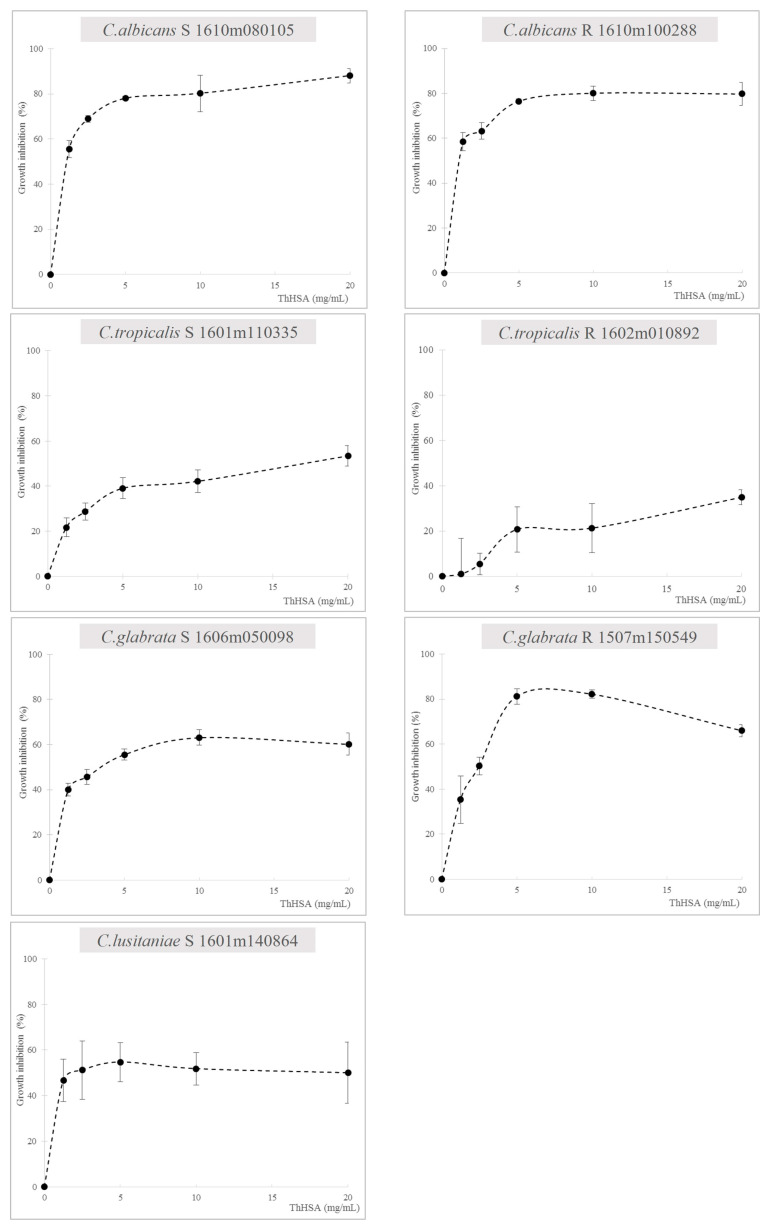
Growth inhibition (%) of ThHSA (0–20 mg/mL) in combating different sensitive and resistant *Candida* spp. were evaluated in triplicate. Data are presented as mean +/− standard deviation.

We then evaluated the activity of ThHSA at increasing concentrations (range: 0–20 mg/mL). There was a dose-dependent effect with peak activity around 5 mg/mL of ThHSA in combating *C. albicans* (S, R), *C. tropicalis* S, *C. glabrata* S, and C. *lusitaniae* S. A complex dose-dependent relationship was noted for both *C. tropicalis* R and *C. glabrata* R growth inhibition (Figure 2). These data argue for the existence of strain-specific effects on each *Candida* strain.

We next determined the inhibitory effect of the antifungal VCZ for the seven *Candida* strains (see Section 4), alone or in combination with ThHSA. At a VCZ concentration of 0.25 µg/mL, we obtained 83.6% and 91.2% of inhibition for *C. albicans* S and R, respectively. For *C. tropicalis* S, at a VCZ concentration of 2.5 µg/mL, we obtained 45.3% of inhibition, whereas for *C. tropicalis* R at 10 µg/mL we obtained 15.7% of inhibition. At a VCZ concentration of 10 µg/mL, 93.3% of inhibition was obtained for *C. glabrata* S, 3.5% of inhibition for *C. glabrata* R, and 89.2% for *C. lusitaniae* (Table 1A,B).

### 2.2. Influence of BSA on the Antimicrobial Activity of Chromogranin A-Derived Peptides

In a preliminary experiment, we compared the antimicrobial activity of BSA against *Candida albicans S* with that of ThHSA and HSA (Figure S4). The structural integrity of BSA was demonstrated by using a hybrid electrospray quadrupole time-of-flight instrument, as detailed in Section 4. The observed masses corresponded to the theoretical value 69,293 Da. BSA (20 mg/mL) also displayed antimicrobial activity against *C. albicans*, with a rate of 77% for growth inhibition. In addition to the intrinsic antimicrobial effect of albumin, we wondered whether it could impact the antimicrobial activities of physiologically available antimicrobial peptides. Bovine serum albumin (BSA) was used because we analyzed the combinations with peptides derived from the natural processing of bovine CgA, CTS (CgA344-364; SSMKLS FRARAYGFRGPGPQL) and CTL (CgA344-358; SSMKLS FRARAYGFR). These two peptides play a crucial role in innate immune activity. They exhibit broad-spectrum antimicrobial activity against bacteria and fungi by disrupting microbial membranes, modulating immune responses, and regulating inflammation [20,27].

Human serum albumin (HSA) shows sequence homology (76%) and structural similarity with bovine serum albumin (BSA) [28]. In addition, it was shown that human, bovine, and ovalbumin showed antimicrobial activity against *C. albicans* cultures, and their effect was dose-dependent [23]. Indeed, in a previous study we reported that BSA (0.4 ng/mL, 1.6 ng/mL) enabled the penetration of CTS (9.6 µg/mL) into *C. albicans*, this positive effect of BSA being related to the conformation of this specific antimicrobial peptide [20].

**Figure 3:**
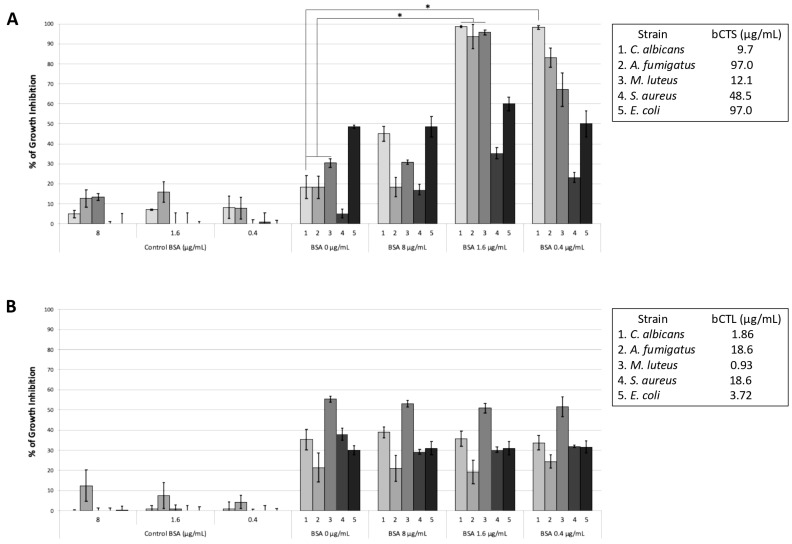
Capability of BSA (0.4 µg/mL to 8 µg/mL) to impact the antimicrobial activities of (**A**) CTS (9.6 µg/mL to 96 µg/mL) and (**B**) CTL against *C. albicans* (S) and also *Aspergillus fumigatus*, *Micrococcus luteus*, *Staphylococcus aureus*, and *Escherichia coli*. The inhibitory rates (%) were evaluated in triplicate. Statistical analyses were obtained using Kruskal–Wallis ANOVA, followed by pairwise comparisons between the indicated groups. * *p*-value < 0.05 was considered statistically significant.3. Discussion.

In the present study we extended our investigations to additional microorganisms. We thus tested the effect of BSA combined with CTS (2 µg/mL) on the growth of *A. fumigatus*, *M. luteus*, *S. aureus*, and *E. coli*. For *A. fumigatus*, low concentrations of BSA (0.4 and 1.6 μg/mL) combined with CTS (9.6 µg/mL) resulted in an increase in the inhibition rate to 83% and 94% compared with that of CTS alone (19%; *p* < 0.05) (Figure 3A). Similarly, for *M. luteus*, low concentrations of BSA (0.4 and 1.6 μg/mL) combined with CTS (12.1 µg/mL) led to inhibition rates of 67% and 96% vs. 30% for CTS alone (*p* < 0.05) (Figure 3A). For *S. aureus* (strain 25923), a resistant Gram-positive bacterium (48 µg/mL bCTS), inhibition rates of 17%, 35%, and 23% were obtained with BSA concentrations of 0.4, 1.6, and 8 μg/mL, respectively, in comparison with an inhibition rate of 5% obtained with CTS alone (Figure 3A). For Gram-negative *E. coli* (strain 25922), BSA concentrations of 0.4, 1.6, and 8 μg/mL combined with 96 µg/mL bCTS led to inhibition rates of 48%, 60%, and 50% in comparison with 50% obtained with CTS alone (Figure 3A). In contrast, no synergistic effect or enhancement of activity was observed for the antimicrobial activities of combinations bCTL-BSA (Figure 3B).

In the following experiments we assessed the ability of ThHSA to impact the low-level action of VCZ in combating *C. tropicalis* S and R, and *C. glabrata* R. For C. *tropicalis* S there was a small, though not significant, increase in inhibition (45.3% to 56.9%) for the combination VCZ (2.5 µg/mL) with ThHSA (5 mg/mL). With one-way ANOVA Kruskal–Wallis multiple comparison, we noted that the addition of ThHSA (1.25 mg/mL to 5 mg/mL) resulted in a significant increase in *C. tropicalis* R growth (*p* = from 0.0119 to 0.0008). When comparing the rate of growth inhibition of ThHSA 2.5 mg/mL with that of the combination ThHSA 1.25 mg/mL with VCZ 2.5 µg/mL, we reached a significant effect, (*p* < 0.05); and for comparison of the growth inhibition of VCZ 2.5 µg/mL with that of the combination ThHSA 2.5 mg/mL with VCZ 2.5 µg/mL, we also obtained a significant impact, (*p* < 0.05) (Table 1A,B).

For *C. glabrata* R, the addition of ThHSA (5 and 10 mg/mL) to VCZ (0.625 µg/mL to 10 µg/mL) induced a significant increase in inhibition growth (3.5% to 65.2%, *p* < 0.0001, with a subsequent decrease in the activity of ThHSA (Table 1B). Similarly, we reported a significant positive effect of ThHSA 10 mg/mL in the combination ThHSA-VCZ compared with VCZ 10 µg/mL alone (*p* = 0.0001), as well as in combination with ThHSA 5 mg/mL and ThHSA 2.5 mg/mL (*p* = 0.0001 and *p* = 0.0002, respectively). Finally, the negative impact of VCZ on the activity of ThHSA was not significant (*p* > 0.99).

We noted the inability of ThHSA to increase the activity of VCZ in combating *C. albicans* R and *C. glabrata* S (Table 1A,B). For *C. albicans* S, the addition of ThHSA (1.25–5 mg/mL) elicited a non-significant increase in the activity of VCZ (0.25 µg/mL) from 83.6% to 95.6% (Table 1A).

Finally, the potential hemolytic effect of ThHSA-VCZ combinations on erythrocyte viability was assessed (Figure S3) as a preliminary measure to check whether albumin induced a hemolytic effect. In contrast with the positive control (isopropanol-1M HCl), none of the tested combinations exhibited a significant effect.

## 3. Discussion

Antibiotic resistance is recognized worldwide as a leading cause of concern in caring for infection [29] on account of both its related morbid mortality and the decreased arrival of new effective antimicrobial drugs on the market. The frequent use of antibiotics has undoubtedly increased antimicrobial resistance, even in patients with COVID-19 [30], thereby leading to therapeutic impasses. *Candida* spp. are frequently involved in a wide array of infections ranging from mucocutaneous injuries to systemic infections. Furthermore, they lay the groundwork for subsequent care-related bacterial infections [31]. Initial antifungal therapy often proves poorly adapted on account of unexpected antifungal resistance, with an accompanying risk of disease spreading. In an attempt to improve current therapeutic approaches to such severe infections, we sought to test whether commercially available ThHSA, which can substitute for HSA and BSA, may be capable of enabling more effective treatment of such infections.

Whilst a few previous papers have noted the involvement of HSA in the growth inhibition of microbes, the usual conclusion reported is that HSA must be associated with other molecules in order to produce this result [18,19,21,22]. In the present study we report, in common with other studies [23], a direct effect of both HSA and ThHSA used alone, which suggests the action of an intrinsic property of albumin. Such an effect is in line with two classes of cell surface binding sites for serum albumin by *C. albicans* [32]. In addition, we underscore that ThHSA and its reference chemical standard (HSA) are frequently, but not always, able to inhibit in an equivalent manner the growth of *Candida* spp. This implies a unique mechanism of action for both drugs linked to the structure of albumin rather than the intervention of either a possible impurity or an intrinsic property of commercially available ThHSA itself. Furthermore, our data suggest for the first time that ThHSA can limit the growth of some *Candida* spp. in humans [13] and this can explain the discrepancies in outcomes reported among patients from different clinical trials [16]. The latter ability of ThHSA is also in line with the decrease in fungi colonization in critically ill patients [13,26]. Nevertheless, it must be emphasized that the growth inhibition of ThHSA is eminently variable according to the strains involved in infection. In addition, the growth inhibitory effect of HSA reaches its maximum at low concentrations, as reported in other studies [33]. This finding is consistent with the known antioxidative properties of HSA, which facilitate the disaggregation of certain proteins and its carrier functions, both of which are essential for optimal activity [34]. Finally, we would draw attention to a paradoxical inefficiency of HSA *in vitro* occurring unexpectedly in our experiments. This indicates the need for the defense mechanisms to be further clarified, and it suggests that ThHSA infusion is not advisable before the sensitivity of *Candida* spp. to HSA has been tested.

In the present study we also confirm that the respective activities of both ThHSA and VCZ, whether taken alone or in combination, inhibit the growth of various, but not all, *Candida* spp. In experiments where VCZ combined with ThHSA (which can itself be linked by ThHSA [35]) was tested for *Candida* spp. growth inhibition, we frequently noticed additive but never synergistic effects with ThHSA. However, in a *C. tropicalis* strain that displayed the highest resistance to VCZ [36], we also reported growth development against all odds. Such a result raises concerns as to the mechanism of action of HSA and/or VCZ for clinical use. It suggests either that HSA is hindering VCZ’s fungicidal power or that VCZ is hindering the intrinsic ability of ThHSA, thereby limiting growth inhibition, or even both. Our result may indicate competing actions of ThHSA and VCZ on the cell membrane with a possible allosteric effect. In any case, these data are in line with (1) *in vivo* data showing that albumin augments the pathogenic potential of *C. glabrata* during the interaction with vaginal epithelial cells [37], (2) data concerning oral mucosa infections caused by *Candida* spp. [38] and, finally, (3) data on the discrepancy between *in vitro* sensitivity of some yeast to antifungal drugs and their in vivo spreading despite *in vitro*-adapted treatment [39,40]. The papers cited on this point report that the pathogenicity of *Candida* spp. may be enhanced with albumin by facilitating the uptake of essential components for *Candida* growth (such as cholesterol or fatty acids). In the present *in vitro* study, these components are constitutive elements of the Sabouraud dextrose broth medium.

Antimicrobial peptides display a potential for killing multi-drug-resistant pathogens without eliciting resistance. Recently it was reported that a synthetic fragment of CgA (CgAN-12) inhibited the level of inflammation factors and promoted the expression of SOCS1, an action that may constitute the mechanism whereby CgAN-12 inhibits *C. albicans* growth and survival [41]. In the second part of our investigation, the pharmacodynamical impact of the interaction of albumin with natural CgA-derived antimicrobial peptides to which HSA is likely exposed in critically ill patients [26] was assessed. These peptides are present in human fluids [42] involved in host defense responses [43,44]. CTS (CgA 344-364) was identified as a catecholamine release-inhibitory peptide and the smaller peptide cateslytin (CTL) (Cga344-358) as displaying a more substantial inhibitory effect on catecholamine release and also on pathogens [43,44]. Our data show that albumin significantly increases the antimicrobial effect of CTS but not CTL on *Candida* spp. growth, confirming previous results in regard to oral candidosis [20]. Thus, we have now confirmed in another model the role of the C-terminal sequence of CTS (GPGPQL) in preventing the penetration of CTS into *C. albicans* in comparison with CTL; and we have further confirmed the relevance of the earlier data obtained by using quartz crystal microbalance (QCM) analysis, which showed that albumin interacts with CTS but not CTL [20].

Moreover, the molecular dynamics simulation of CTS leads to a beta strand–loop–beta strand structure [45]. R10, A11, and Y12 contribute to helix and, in contrast, F7, R8, A9, F14, R15, G16, P17, and G18 contribute to the antiparallel beta sheet structure. Such a folding structure facilitates an amphipathic conformation with positively charged and hydrophobic faces [45]. In addition, multidimensional solution NMR and circular dichroism spectroscopies have confirmed that CTS adopts alpha-helical conformations between S6 and Y12 in the presence of dodecylphosphocholine micelles [46]. Furthermore, proton-decoupled (15)N solid-state NMR spectroscopy of sequences labeled with (15)N and reconstituted into oriented lipid bilayers indicates that this domain is aligned with a strongly tilted planar alignment. Proton-decoupled (31)P NMR spectra of the same samples are indicative of conformational and/or orientational heterogeneity at the level of the lipid bilayer head groups on account of the presence of CTS. The sequence and three-dimensional structure of CTS exhibit homologies with penetratin, which is suggestive that they both enter the cells by related mechanisms to target internal structures [47]. In contrast, CTL is non-structured in solution but is converted into an antiparallel beta structure and forms aggregates at the surface of negatively charged bacterial membranes and membrane pore formation in the domains containing ergosterol [48]. Cationic antimicrobial peptides such as CTS or CTL may thus bind with their hydrophobic parts to drug site II of HSA [49]. For bovine and human sequences of CTS (RSMRLSFRARGYGFRGPGPQL and SSMKLSFRARAYGFRGPGPQL, respectively), the hydrophobic domain is highly conserved and contains M3, L5, F7, Y12, and F14. It corresponds to the CTL sequence (RSMRLSFRARGYGFR) that is able to rapidly penetrate *C. albicans*. However, this non-structured domain is unable to interact with albumin [20]. In contrast, the structure of CTS allows the interaction with albumin by the C-terminal domain (GPGPQL), which may explain how BSA improves the penetration of CTS into *Candida* spp.

Finally, we cannot rule out the existence of other mechanisms for the interaction between ThHSA and CTS, since the chemical structure of CTS could be modified by the binding to BSA to prevent its association with the *Candida* membrane while allowing its penetration. This may also explain why the combination of BSA with the peptide CTS kills *A. fumigatus* (Figure 3).

In addition, the role of CTS and CTL as immunomodulators of immune cells has previously been reported [47,50]. Indeed, treatment with CTS induces a decrease in pro-inflammatory cytokines IL-6, IL1 β, and TNFα [50], and these effects may be enhanced by albumins. Furthermore, since CTL (but not CTS) is resistant to proteases produced by *S. aureus*, its primary and secondary structures are important for its antibacterial activity [51].

All these data are in line with those from a randomized clinical trial in which the use of the continuous infusion of low doses of 4% ThHSA in non-selected critically ill patients with shock resulted in a significant decrease in the occurrence of both colonization and care-related infections when compared with the standard intermittent 20% ThHSA treatment [26].

## 4. Materials and Methods

### 4.1. *Candida* Strains

Various strains of *C. albicans* S/R (1610m080105/04 and 1610m100288), *C. tropicalis* S/R (1601m110335 and 1602m010892), *C. glabrata* S/R (1606m050098 and 1507m150549), and *C. lusitaniae* S (1601m140864/02) were kindly provided by the Institute of Parasitology and Tropical Pathology, Strasbourg, France. These strains were characterized by their susceptibility and resistance to Amphotericin B, Fluconazole, VCZ, and Caspofungin using the Etest method (AB BIODISK, Solna, Sweden) [20]. For the Etest, 90 mm diameter plates containing agar at a depth of 4.0 mm were used. The agar surface was inoculated by using a nontoxic swab dipped in a cell suspension adjusted spectrophotometrically to the turbidity of a 0.5 McFarland standard. After excess moisture was absorbed into the agar and the surface was completely dry, an Etest strip was applied to each plate. The plates were incubated at 35 °C and read at 48 h. The MIC was read at the lowest concentration at which the border of the elliptical inhibition zone intercepted the scale on the strip.

### 4.2. Characterization of Albumins (HSA and ThHSA)

Human and bovine serum albumin (HSA, BSA) were obtained from Euromedex (Souffelweyersheim, France), while therapeutic human serum albumin (ThHSA; Vialebex 200 mg/mL) was purchased from LFB (Les Ulis, France). Both HSA and ThHSA were analyzed using a Dionex HPLC system (Ultimate 3000, Sunnyvale, CA, USA) equipped with a Vydac 208TP C8 column (2.1 × 150 mm) and a pre-column Vydac 208TP (7.5 × 2.1 mm), following previously established experimental conditions [20]. The structural integrity of HSA, BSA, and ThHSA was assessed using a hybrid electrospray quadrupole time-of-flight mass spectrometer (Synapt G2 HDMS, Waters, Manchester, UK) coupled with an automated chip-based nanoelectrospray device (Triversa Nanomate, Advion Biosciences, Ithaca, NY, USA), operated in positive ion mode.

### 4.3. Preparation of Voriconazole and MICs for Antibiotics

VCZ laboratory-grade standard powder was supplied by Pfizer (New York, NY, USA). Solutions were freshly prepared according to the manufacturer’s instructions immediately prior to use. Determination of minimal inhibitory concentrations (MICs) of Amphotericin B, Fluconazole, VCZ, and Caspofungin were determined for the *Candida* strains using the Etest method, as per the manufacturer’s protocol and established guidelines.

### 4.4. Growth Inhibition Assays of HSA, ThHSA, and Voriconazole Against Candida Strains

The antifungal activities of HSA, ThHSA, and VCZ were evaluated in triplicate using the macrodilution method M27-A2, as approved by the National Committee for Clinical Laboratory Standards. *C. albicans* cells were pre-cultured in Sabouraud dextrose broth supplemented with tetracycline (10 µg/mL) and cefotaxime (0.1 µg/mL). Spores were suspended to an initial absorbance of A620 nm = 0.001, and assays were conducted in 96-well plates (Sarstedt AG and Co., Nümbrecht, Germany). Aqueous solutions of HSA and ThHSA (200 mg/mL, 20%; 100 mg/mL, 10%; 50 mg/mL, 5%; 25 mg/mL, 2.5%; 12.5 mg/mL, 1.25%) were prepared, and 10 µL of each solution was incubated with 90 µL of the *Candida* suspension at 37 °C for 24 h under continuous agitation. For positive controls, 10 µL of VCZ (10 µg/mL) was used to ensure complete growth inhibition, while 10 µL of distilled water served as the negative control, representing zero growth inhibition. Fungal growth was quantified by measuring absorbance at 620 nm, and all experiments were performed in triplicate. The MIC was defined as the lowest concentration of the compound that inhibited visible fungal growth under the experimental conditions. VCZ (0–10 µg/mL) was tested against the various *Candida* strains, and MIC values were determined following previously published methodologies.

### 4.5. Analysis of the Antimicrobial Activity of ThHSA in Combination with Voriconazole

To evaluate potential effects, combinations of ThHSA and VCZ were tested against *Candida* strains. Specifically, 10 µL of ThHSA (0–10 mg/mL) was combined with 10 µL of VCZ (0–10 µg/mL) and incubated under the same conditions described above. Each experiment was conducted in triplicate, following established protocols, to assess whether ThHSA enhances the antifungal activity of VCZ.

### 4.6. Preparation and Characterization of Synthetic CgA-Derived Peptides

CTS and CTL were provided by Pepmic (Suzhou, China). Their purity was assessed using reverse-phase (RP) HPLC, employing a Dionex HPLC system (Ultimate 3000, Sunnyvale, CA, USA) with a Vydac 208 TP C8 column (2.1 × 150 mm) and a Vydac 208 TP 14 pre-column (7.5 × 2.1 mm) (Vydac, AIT France, Houilles, France) according to the previously described method [20].

### 4.7. Antimicrobial and Antifungal Assays for Albumin and CgA-Derived Peptides

The antimicrobial activity of CTS and CTL was tested on Gram-positive *S. aureus* 25923 (ATCC), Gram-negative *E. coli* 25922 (ATCC), and *M. luteus* B199. Bacteria were pre-cultured aerobically at 37 °C in Mueller–Hinton broth (MHB) medium or lysogeny broth (LB) medium for *E. coli*. After 24 h incubation on agar plates, a colony from each strain was transferred to 10 mL MHB medium and incubated for 24 h to reach the stationary growth phase. The bacteria were then suspended in MHB to obtain an optical density (OD) of 0.001 at 620 nm, corresponding to the mid-logarithmic phase. The antimicrobial effects of CTS and CTL solutions (10 µL each) were tested in a 96-well plate (Sarstedt AG and Co., Nümbrecht, Germany) against *M. luteus* B199 (12 µg/mL CTS, 0.93 µg/mL CTL), *S. aureus* 25923 (48 µg/mL CTS, 18.6 µg/mL CTL), and *E. coli* 25922 (96 µg/mL CTS, 3.72 µg/mL CTL) in 90 µL of diluted bacterial solutions. Aqueous solutions of BSA (8, 1.6, and 0.4 µg/mL) were also tested. Tetracycline (10 µg/mL) and cefotaxime (0.1 µg/mL) were used as positive controls. Microbial growth was assessed by measuring absorbance at OD 620 nm after 24 h of incubation at 37 °C. The control cultures without peptides/albumin showed 0% inhibition, while cultures with antibiotics were considered to have 100% inhibition. Each assay was conducted in triplicate. The antifungal activities of the molecules were tested on the filamentous fungus *A. fumigatus* and the yeast *C. albicans*. For *A. fumigatus*, spores were suspended in potato dextrose broth (PDB) medium supplemented with 10 µg/mL tetracycline and 0.1 µg/mL cefotaxime. The fungal growth was tested in a 96-well plate, incubated at 37 °C for 24 h. The antimicrobial effects of CTS and CTL solutions (10 µL each) were tested against *C. albicans* (9.6 µg/mL CTS, 1.86 µg/mL CTL) and *A. fumigatus* (96 µg/mL CTS, 18.6 µg/mL CTL) in 90 µL of diluted bacterial solutions. Aqueous solutions of BSA (8, 1.6, and 0.4 µg/mL) were also tested. The MTT assay was used to measure fungal metabolic activity, with absorbance at 540 nm indicating growth. The control cultures showed 0% inhibition, while cultures with 1 µg/mL VCZ showed 100% inhibition. Similarly, for *C. albicans*, spores were suspended at 10^4^ spores/mL in Sabouraud medium with a starting OD of 0.001. The same concentrations of CTS and CTL were tested, and the MTT assay was used to evaluate fungal growth. BSA was tested under similar conditions as previously reported.

### 4.8. Hemolysis Assay

The cytotoxicity of the ThHSA-VCZ combination was assessed using a hemolysis assay, conducted according to previously published methods [20]. Briefly, erythrocytes were washed with 0.15 M sodium chloride and resuspended in PBS (pH 7.4) to achieve a concentration of 10^8^ cells/mL. For controls, isopropanol-1M HCl was used to induce complete hemolysis (positive control), while 10 µL of PBS in 90 µL of erythrocyte suspension served as the negative control. To assess toxicity, 10 µL of ThHSA (20 mg/mL) and VCZ (0.5 µg/mL) were added to 90 µL of erythrocyte suspension followed by incubation at 37 °C for 40 min. Hemolytic activity, measured by hemoglobin release at 420 nm, was assessed to ensure the safety of the combined treatment on mammalian cells.

### 4.9. Statistical Analysis

Data are presented as mean ± standard deviation (SD). Where appropriate, non-parametric statistical analyses were performed using Kruskal–Wallis ANOVA, followed by pairwise comparisons between groups. All analyses were conducted using GraphPad Prism 5.0 software (GraphPad Software Inc., San Diego, CA, USA). A *p*-value < 0.05 was considered statistically significant.

## 5. Conclusions

The present study suggests the use of commercially available ThHSA as a complementary tool for the care of some *Candida* spp. infections in humans, provided that some precautions are fully respected. First, HSA sensitivity of *Candida* spp. should be assessed *in vitro* in relation to isolated strains before any ThHSA infusion. Second, when targeting a microbe, there is no need for standard or high doses of ThHSA, but it is crucial for efficient pharmacological effects that HSA be present in vivo in its non-oxidized form [13]. Albumins thus represent a conceivable tool for improving defense in the fight against some microbes, even as a preventive option. Finally, albumin can be considered as a help in the enhancement of some endogenous antimicrobial peptides for proper and effective care of some, though not all, human infections.

## Figures and Tables

**Table 1:** (**A**) Capability of ThHSA to impact the antimicrobial activity of VCZ in combating sensitive (S) *Candida* spp. The inhibitory rates (%) were evaluated in triplicate and data are presented as mean. Values in bold correspond to VCZ-ThHSA combinations showing an increased inhibitory rate compared with VCZ alone. --, non-tested concentrations. (**B**) Capability of ThHSA to impact the antimicrobial activity of VCZ in combating resistant (R) *Candida* spp. The inhibitory rates (%) were evaluated in triplicate and data are presented as mean. Values in bold correspond to VCZ-ThHSA combinations showing an increased inhibitory rate compared with VCZ alone, whereas values in bold and italic correspond to VCZ-ThHSA combinations showing an enhanced growth relative to VCZ.

(A)																			
		*C. albicans* (S)				*C. tropicalis* (S)				*C. glabrata* (S)					*C. lusitaniae* (S)				
	**ThHSA** **(mg/mL)**	0.0	1.25	2.50	5.00	0.0	1.25	2.50	5.00	0.0	1.25	2.50	5.00	10.00	0.0	1.25	2.50	5.00	10.00
**VCZ** **(μg/mL)**																			
0.0000		0.0	55.5	68.9	77.9	0.0	21.7	28.7	39.1	0.0	38.9	45.6	55.5	63.1	0.0	46.8	51.2	54.7	51.8
0.0156		6.6	**31.9**	**56.5**	**72.6**	--	--	--	--	--	--	--	--	--	--	--	--	--	--
0.0312		19.8	**34.8**	**64.3**	**73.5**	--	--	--	--	--	--	--	--	--	--	--	--	--	--
0.0625		18.7	21.9	**73.9**	**75.3**	--	--	--	--	--	--	--	--	--	--	--	--	--	--
0.1250		44.8	50.1	**67.1**	**70.6**	--	--	--	--	--	--	--	--	--	--	--	--	--	--
0.1560		--	--	--	--	10.1	7.4	**29.3**	**38.7**	--	--	--	--	--	--	--	--	--	--
0.2500		83.6	97.9	99.8	95.6	--	--	--	--	--	--	--	--	--	--	--	--	--	--
0.3120		--	--	--	--	7.6	7.4	**31.3**	**42.1**	--	--	--	--	--	--	--	--	--	--
0.6250		--	--	--	--	8.2	10.9	**34.5**	**40.4**	8.2	**63.7**	**44.5**	**52.1**	**58.6**	−25.1	**29.3**	**57.1**	**41.7**	**64.9**
1.2500		--	--	--	--	10.8	8.4	**20.4**	**42.3**	30.2	**55.1**	**42.3**	**53.1**	**61.9**	34.1	35.5	**50.7**	**50.9**	**72.7**
2.5000		--	--	--	--	45.3	52.5	51.3	**56.9**	60.5	49.9	50.2	57.8	64.7	68.6	68.5	57.3	70.7	65.5
5.0000		--	--	--	--	--	--	--	--	84.1	75.2	77.1	86.6	84.9	85.9	97.7	98.4	97.4	95.8
10.0000		--	--	--	--	--	--	--	--	93.3	89.0	90.0	92.9	93.4	89.2	98.9	100	100	100
**(B)**																			
		***C. albicans* (R)**				***C. tropicalis* (R)**							***C. glabrata* (R)**						
	**ThHSA** **(mg/mL)**	0.0		1.25	2.50	5.00		0.0	1.25	2.50	5.00	10.00	0.0		1.25	2.50		5.00	10.00
**VCZ** **(μg/mL)**																			
0.0000		0.0		58.5	63.2	76.5		0.0	1.0	5.4	20.7	21.3	0.0		35.3	50.2		81.1	82.2
0.0156		12.4		**58.7**	**71.4**	**79.6**		--	--	--	--	--	--		--	--		--	--
0.0312		11.1		**59.5**	**72.5**	**79.3**		--	--	--	--	--	--		--	--		--	--
0.0625		9.5		**57.2**	**77.5**	**77.8**		--	--	--	--	--	--		--	--		--	--
0.1250		33.8		**59.5**	**72.5**	**79.8**		--	--	--	--	--	--		--	--		--	--
0.2500		91.2		93.9	97.6	90.9		--	--	--	--	--	--		--	--		--	--
0.6250		--		--	--	--		18.6	***−55.6***	***−66.5***	***0.0***	***0.8***	−4.6		**58.7**	**40.8**		**43.2**	**43.6**
1.2500		--		--	--	--		16.6	***−54.4***	***−63.8***	***−10.0***	**7.6**	−4.5		**59.5**	**40.2**		**43.1**	**43.9**
2.5000		--		--	--	--		18.4	***−60.3***	***−59.1***	***1.0***	14.6	−0.1		**57.2**	**34.9**		**45.6**	**49.2**
5.0000		--		--	--	--		17.4	***−64.9***	***−52.6***	***1.6***	14.0	−3.0		**59.5**	**44.6**		**36.9**	**50.1**
10.0000		--		--	--	--		15.7	***−55.5***	***−55.4***	***1.4***	21.8	−3.5		**43.9**	**60.3**		**55.2**	**65.2**

## Data Availability

The data are stored at the Department of Biomaterials and Bioengineering, Institut National de la Santé et de la Recherche Médicale (INSERM), Unité Mixte de recherche (UMR) S 1121, Strasbourg, France. The experimental data that support the findings of this study will be made available upon formal request to the corresponding authors and consequent approval of the proposal by the INSERM-UMRS 1121 research group. Requests should be sent to francesco.scavello@humanitasresearch.it and marie-helene.metz@inserm.fr.

## Supplementary Materials

The following supporting information can be downloaded at: https://www.mdpi.com/article/10.3390/antibiotics14100974/s1, Figure S1: The experimental flowchart; Figure S2: A comparative HPLC of Th HSA and HSA. Peaks were detected at an absorbance of 214 nm; B, hybrid electrospray quadrupole time-of-flight mass spectrometry of HSA (69240 Da) and Th HSA (66285 Da); Figure S3: Hemolytic assay of erythrocytes with ThHSA (20 mg/mL) combined with VCZ (0.5 µg/mL); *p* < 0.001; Figure S4: Antimicrobial activities of ThHSA and HSA (20 mg/mL) in combating sensitive (S) and resistant (R) *Candida* spp. Antimicrobial activity of BSA (20 mg/mL) was added for sensitive *C. albicans* (see black arrow). Microbial growth was quantified by measuring absorbance at 620 nm. Positive control corresponds to VCZ and negative control to MilliQ water. The inhibitory rates (%) were evaluated in triplicate. Data are presented as mean +/− standard deviation.

## Abbreviations

The following abbreviations are used in this manuscript:

A620 nm	American Type Culture Collector
Alb	Albumin
BSA	Bovine Serum Albumin
bCTS	Bovine Catestatin
bCTL	Bovine Cateslytin
HSA	Human Serum Albumin
HPLC	High-Performance Liquid Chromatography
ICU	Intensive Care Unit
MS	Mass Spectrometry
PDB	Potato Dextrose Broth
QCM	Quartz Crystal Microbalance
Th Alb	Therapeutic Albumin
Th HSA	Therapeutic Human Serum Albumin
VCZ	Voriconazole
