# Supplementary material for: Insight into the Roles of Albumin—Alone and in Combination with Either Voriconazole or Antimicrobial Peptides Derived from Chromogranin A—In the Growth of Different Microbial Species
Source: Antibiotics (Basel). 2025 Sep 26;14(10):974. doi: 10.3390/antibiotics14100974 (PMC12561135; doi:10.3390/antibiotics14100974)
Supplement: Supplementary file 1 [file antibiotics-14-00974-s001.zip › antibiotics-3787633-supplementary.pdf]

Figure S1

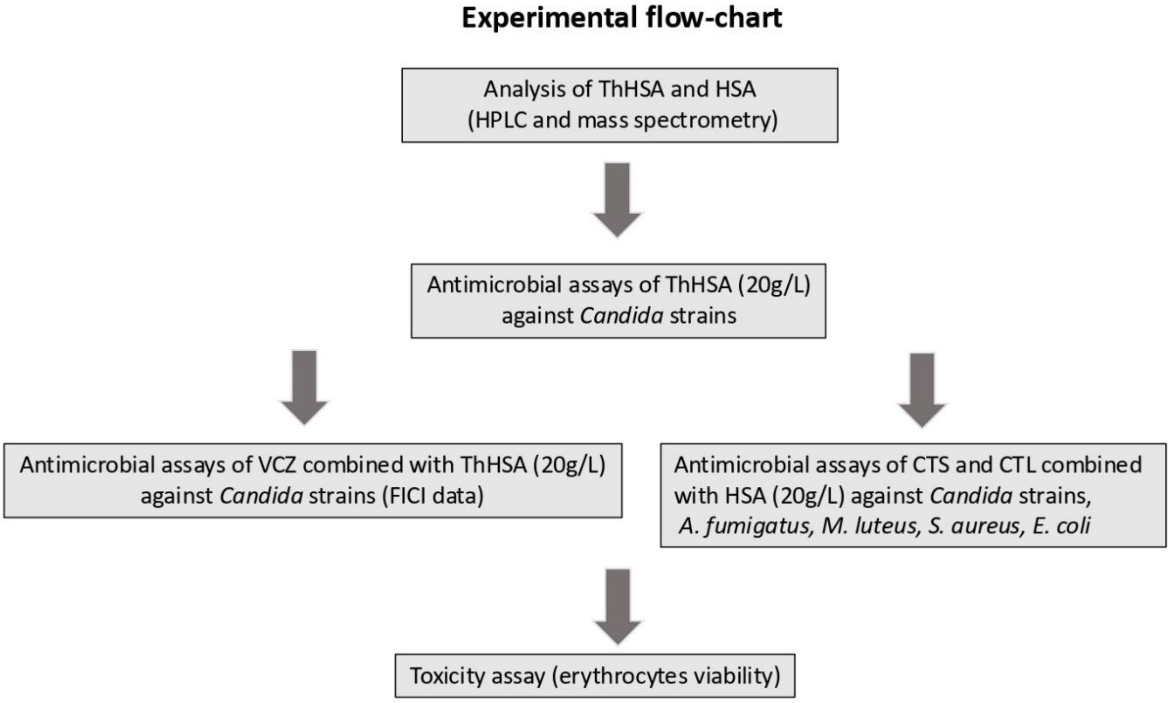

Figure S1. The experimental flow-chart.

Figure S2

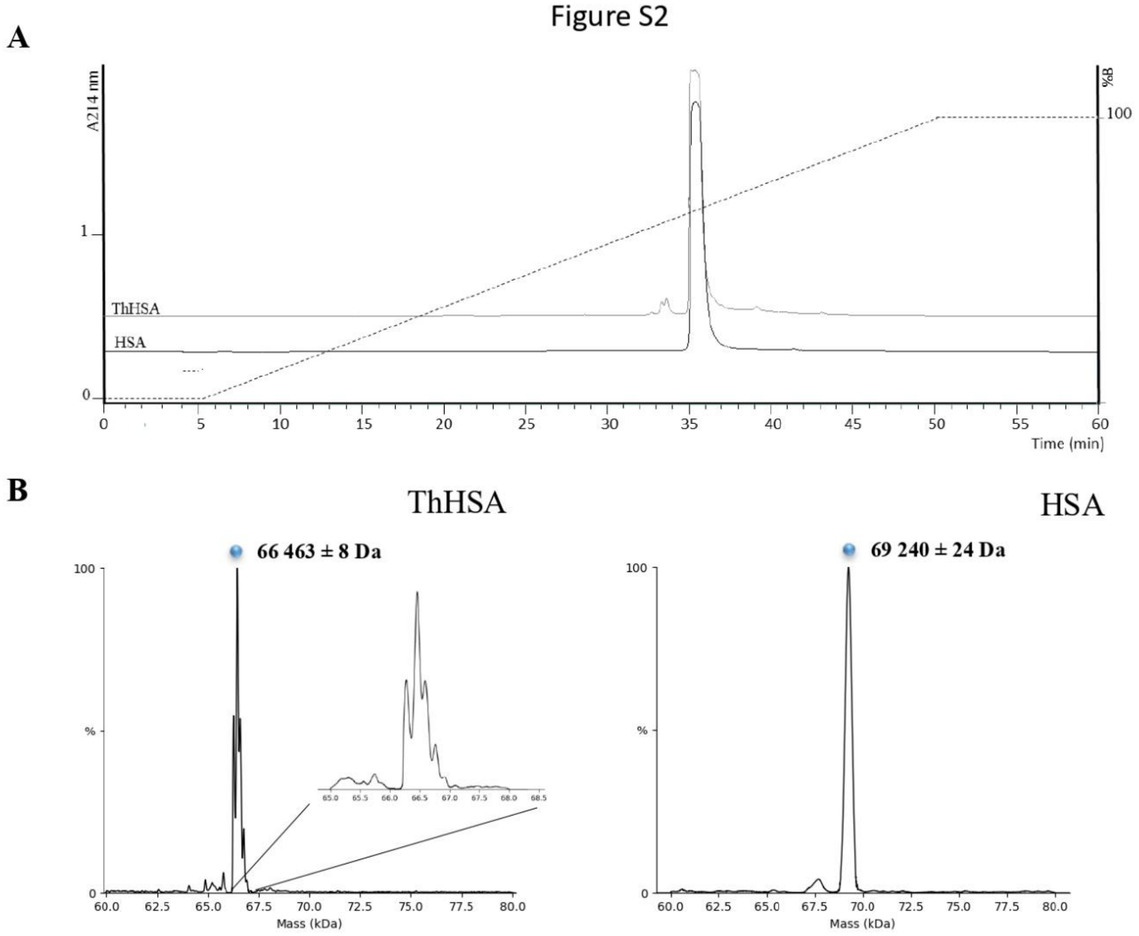

Figure S2. A comparative HPLC of Th HSA and HSA. Peaks were detected at an absorbance of 214 nm; B, hybrid electrospray quadrupole time of flight mass spectrometry of HSA (69240 Da) and Th HSA (66285 Da);.

Figure S3

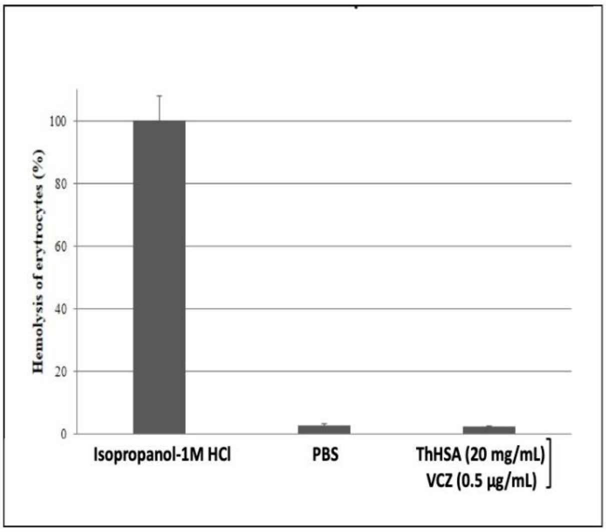

Figure S3. Hemolytic assay of erythrocytes with ThHSA (20 mg/mL) combined with VCZ (0.5 µg/mL); $p<0.001$.

Figure S4

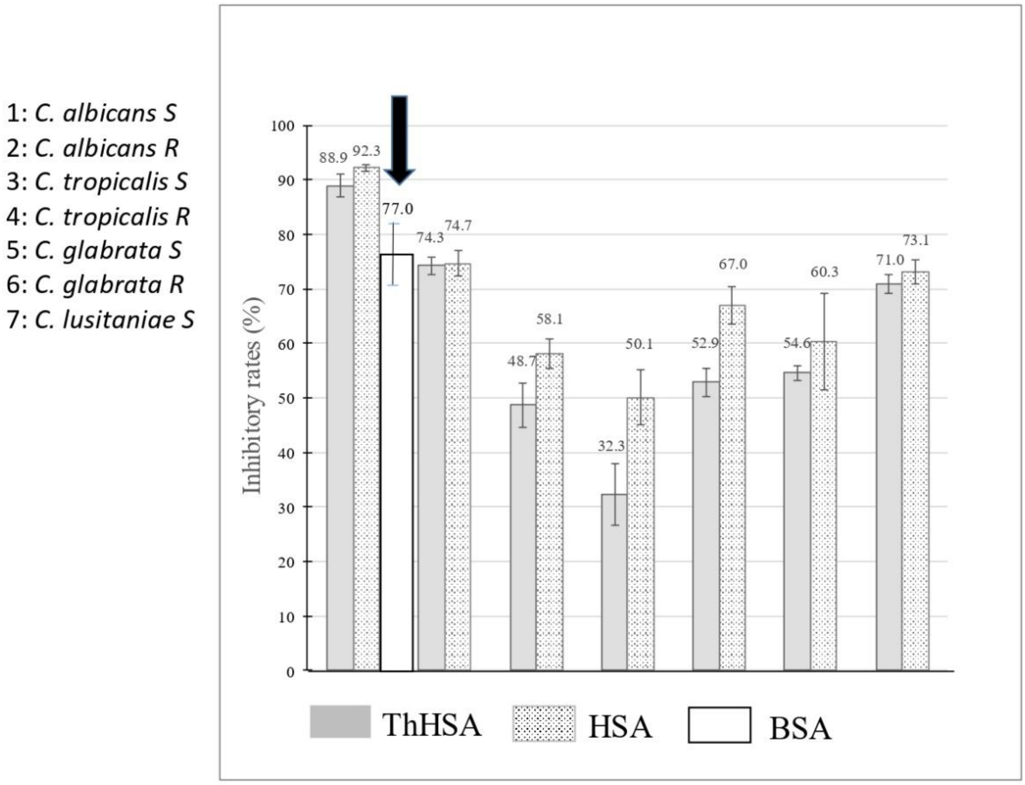

Figure S4: Antimicrobial activities of ThHSA and HSA (20 mg/mL) in combating sensitive (S) and resistant (R) Candida spp. Antimicrobial activity of BSA (20 mg/ml) was added for sensitive C. albicans (see black arrow). Microbial growth was quantified by measuring absorbance at 620 nm. Positive control corresponds to VCZ and negative control to milliQ water. The inhibitory rates (%) were evaluated in triplicate. Data are presented as mean +/- standard deviation.
